# Should policy makers trust composite indices? A commentary on the pitfalls of inappropriate indices for policy formation

**DOI:** 10.1186/s12961-021-00702-4

**Published:** 2021-03-22

**Authors:** Matthias Kaiser, Andrew Tzer-Yeu Chen, Peter Gluckman

**Affiliations:** 1 The Centre for the Study of the Sciences and the Humanities (SVT), University of Bergen, Bergen, Norway; 2 Koi Tū: The Centre for Informed Futures, The University of Auckland, Auckland, New Zealand

**Keywords:** Pandemic preparedness, Composite indicators, Science-for-policy, Decision tools, Heuristics, Scientific uncertainty

## Abstract

**Background:**

This paper critically discusses the use and merits of global indices, in particular, the Global Health Security Index (GHSI; Cameron et al. https://www.ghsindex.org/#l-section--map) in times of an imminent crisis, such as the current pandemic. This index ranked 195 countries according to their expected preparedness in the case of a pandemic or other biological threat. The coronavirus disease 2019 (Covid-19) pandemic provides the background to compare each country's predicted performance from the GHSI with the actual performance. In general, there is an inverted relation between predicted versus actual performance, i.e. the predicted top performers are among those that are the worst hit. Obviously, this reflects poorly on the potential policy uses of this index in imminent crisis management.

**Methods:**

The paper analyses the GHSI and identifies why it may have struggled to predict actual pandemic preparedness as evidenced by the Covid-19 pandemic. The paper also uses two different data sets, one from the Worldmeter on the spread of the Covid-19 pandemics, and the other from the International Network for Government Science Advice (INGSA) Evidence-to-Policy Tracker, to draw comparisons between the actual introduction of pandemic response policies and the corresponding death rate in 29 selected countries.

**Results:**

This paper analyses the reasons for the poor match between prediction and reality in the index, and mentions six general observations applying to global indices in this respect. These observations are based on methodological and conceptual analyses. The level of abstraction in these global indices builds uncertainties upon uncertainties and hides implicit value assumptions, which potentially removes them from the policy needs on the ground.

**Conclusions:**

From the analysis, the question is raised if the policy community might have better tools for decision-making in a pandemic. On the basis of data from the INGSA Evidence-to-Policy Tracker, and with backing in studies from social psychology and philosophy of science, some simple heuristics are suggested, which may be more useful than a global index.

## Significance statement

The paper “Should policy makers trust composite indices?—A commentary on the pitfalls of inappropriate indices for policy formation” addresses an issue of potential practical impact of constructing a global index as a guide to policy. Global indices are produced in several fields, but the paper critically analyses the Global Health Security Index of October 2019 in particular. While the index purports to predict the potential performance of countries during the outbreak of a pandemic, the actual performances during Covid-19 were largely the opposite of these predictions. The reasons for this are analysed, and it is suggested that simple heuristics might do a better job for policy design. The paper concludes with some skepticism on the practical uses of global indices in general.

## Background

Why would anyone want to construct a global composite index of anything? The standard answer is that it would provide a useful tool for policy design and decision-making. In theory, the composite score is easier to understand than a complex concept such as wellbeing or sustainability because it provides a quantified measure. The next question is then: how can users know if the index is good and useful? Tentatively, we might suggest that the index has value if using it leads to better policies and decisions than would have been the case without it. However, most global composite indicators are never really put to the test, because performance is normally not directly measurable. We surmise that the case of the Global Health Security Index (GHSI) [1] may be an exception. This index was published in October 2019 after 2.5 years of research and contained a ranking of 195 countries with their associated scores indicating their preparedness for global epidemics and pandemics. The GHSI aimed to be a key resource in the “face of increasing risks of high-consequence and globally catastrophic biological events in light of major gaps in international financing for preparedness” [1]. The developers “believe that, over time, the GHS Index will spur measurable changes in national health security” and sought to “illuminate preparedness and capacity gaps to increase both political will and financing to fill them at the national and international levels” [1]. It utilized 140 questions organized in six categories, 34 indicators and 85 sub-indicators, all constructed from open-source information. Out of a total possible score of 100, the average for these countries was 40.2, ranging between 83.5 and 16.2. Fewer than 7% of the countries are ranked as being able to effectively prevent the emergence or release of pathogens.

## Methods

Our guiding question was: How accurate was the GHSI? To answer this, we compared two data sets: the Worldometer data on the spread of the coronavirus disease 2019 (Covid-19) pandemic and the International Network for Government Science Advice (INGSA) Evidence-to-Policy Tracker data, both reduced to 29 selected countries based on gross household incomes. The bulk of the paper then analyses the GHSI and identifies challenges for why it may not have been well suited for predicting performance in pandemic response, before demonstrating the power of a simple heuristic in the Conclusion.

Less than half a year after the publication of the GHSI, the novel severe acute respiratory syndrome coronavirus 2 (SARS-CoV-2) led to the Covid-19 pandemic. This provides the possibility to compare the previous assessment from the index with the actual performance of countries’ health systems. Firstly, we can see how the GHSI ranked countries into three levels of preparedness. The United States of America and United Kingdom top the index at rank 1 and 2 (scoring 83.5 and 77.9, respectively), with Sweden (72.2), South Korea (70.2) and France (68.1) with high rankings at 7, 9 and 11, respectively. Then there are countries like Germany (66.0), Spain (65.9), Norway (64.9), Italy (56.2), New Zealand (54.0) and others which are placed in the middle category of preparedness, apparently not so well prepared. Brazil (59.7, rank 22) is ranked slightly better than Singapore (58.7, rank 24). Mongolia is at least above the average, with a score of 49.5, while Jamaica (29.0, rank 147) and Fiji (25.7, rank 168) are among those ranked as very poorly prepared.

## Results

For those who have been watching the global spread of Covid-19, the incongruities between the GHSI rankings and the case numbers in each country will be obvious. June 2020 data from the Worldometer website [https://www.worldometers.info/coronavirus/] show the opposite of what we might expect from the GHSI rankings, with the United States of America, United Kingdom, Sweden and Brazil being the worst hit countries (a trend that continued later) and other countries, namely Germany, Norway, Singapore, New Zealand and Vietnam, surpassing expectations derived from the index. This is certainly also the case for Jamaica and Fiji, which have virtually eliminated Covid-19, but were ranked among the least prepared. Two of the best performing economies (in June 2020), Taiwan and Hong Kong, are not even included in the global index. We do note that there are some limitations with the use of data aggregators like Worldometer and potential issues around the reliability of testing in some countries, but we feel that these data are suitable enough for demonstrating the magnitude of the problem. Table 1 shows a comparison of 29 selected countries, chosen on the basis of gross household income. This subset was chosen as those with the highest household incomes to focus on countries where resource limitations in the health care system would not be the primary determinant. A small rank difference indicates a close estimate between GHSI and reality, while a large rank difference indicates a big gap between expectation and actual performance. Of the 178 countries, only 20 had a Worldometer ranking within 20 rank positions of their ranking on the GHSI.

**Table 1:** A selection of 29 countries with the highest gross household income [2]

Country	GHSI rank	GHS score	Worldometer rank	Deaths per million people	Rank difference
United Kingdom	2	77.9	176	618	174
United States of America	1	83.5	171	359	170
Netherlands	3	75.6	170	354	167
Sweden	7	72.1	173	489	166
Canada	5	75.3	166	218	161
France	11	68.2	172	453	161
Spain	15	65.9	175	580	160
Belgium	19	61.0	178	834	159
Denmark	8	70.4	155	103	147
Ireland	23	59.0	169	346	146
Germany	14	66.0	158	106	144
Finland	10	68.7	149	59	139
Slovenia	12	67.2	143	52	131
Austria	26	58.5	152	76	126
Norway	16	64.6	139	45	123
Luxembourg	67	43.8	162	176	95
Kuwait	59	46.1	150	71	91
Czech Republic	42	52.0	131	31	89
Saudi Arabia	48	49.3	129	30	81
Israel	54	47.3	133	33	79
Republic of Korea	9	70.2	66	5	57
Japan	21	59.8	78	7	57
Australia	4	75.5	59	4	55
Qatar	82	41.2	123	28	41
Bahrain	88	39.4	124	28	36
Singapore	24	58.7	57	4	33
New Zealand	36	54.0	62	4	26
Malta	97	37.3	114	20	17

The rankings are reassigned out of 178, based on the countries in both the GHSI and Worldometer data sets as of 16 June 2020. The table is sorted by the rank difference (Worldometer Rank based on the number of deaths per million people—GHSI)

*GHSI* Global Health Security Index

Quantifying the expected and actual performance data for these 29 countries invites us to look more deeply into why the differences were so large. It is not always obvious what should count as performance data—one might pick the number of infections per million inhabitants, or as we have, the number of deaths per million people. We think it is generally desirable to avoid a high death rate. What we have not done is to break down the total GHSI score into its six subcategories, which would somewhat complicate the picture.^1^ These subcategories in the GHSI report are:

1. Prevention of the emergence or release of pathogens
2. Early detection and reporting of epidemics of potential international concern
3. Rapid response to and mitigation of the spread of an epidemic
4. Sufficient and robust health system to treat the sick and protect health workers
5. Commitments to improving national capacity, financing and adherence to norms
6. Overall risk environment and country vulnerability to biological threats.

Any single country will score differently on these subcategories, such that the GHSI claimed to provide more detailed information on where to act in order to improve general preparedness. We surmise that this might indeed hold true for at least some of the long-term policy improvements in those countries. Yet, the total score is what is used for international comparison and ranking, and is what conveys the most weight in political discussion. In late February, US President Donald Trump cited the GHSI to argue that the United States of America was well prepared for Covid-19, saying “the United States, we’re rated No. 1” [3]. And it is here that the discrepancy arises most clearly with actual performance.

In the subsequent section, we discuss the challenges with the GHSI, and in the conclusion we present a simpler heuristic that could provide a more direct explanation for the success (or not) of pandemic responses.

## Discussion and literature review

We want to ask why was the index so wrong?

There have already been several assessments of how the GHSI fared in the light of Covid-19 [4–7]. All assessments noted significant shortcomings, and based on data from Worldometer, several noted the reversal of relationships. Razavi et al. [6] questions the wisdom of the ranking system: “ranking countries based on weighted scores across indicators that are scored variably and are not directly comparable with one another is problematic”. These authors based this assertion on their criticism that the scoring system is not consistent (some indicators score either 0 or 100, while others use the whole range), the use of weightings is arbitrary and the inclusion of some indicators, such as urbanization, are of questionable validity. Chang and McAleer [7] extended the analysis of GHSI by adding other approaches to quantify mean values (adding to the arithmetic mean used in GHSI, the geometric and harmonic mean values) and saw positive potential in the GHSI, but stressed the significant differentiation in the six indicators: “Rapid Response and Detection and Reporting have the largest impacts” [7]. They also commented on the implicit political bias: “As part of China, Hong Kong was not included in the GHS Index as a country, while Taiwan was not included undoubtedly for political reasons” [7]. Abbey et al. [5] observe the incongruity between prediction and outcome, and stress that political leadership and previous experiences with epidemics should be counted as a crucial factor for preparedness, and therefore be included in the future improvements of the GHSI.

With the partial exception of the Aitken et al. critique [4], most of the criticism focusses on the technical aspects of constructing the composite index, in particular when it comes to combining subcategories to create a total score assigned to the individual countries. Abbey et al. [5] seem to find the main shortcoming in incorrect weighting and incomplete definition of the relevant categories. It is noteworthy, though, that they seem to find pragmatic policy value in various subcategories, thereby putting aside the problem of how these were constructed from a large number of indicators and sub-indicators.

Given the extreme discrepancy between expected and actual performance for most countries, one must ask if this failure might be due to a deeper inherent weakness in the underlying concept of the index, or to other contextual factors. For instance, could underperformance simply be due to political decision makers not utilizing their countries’ capabilities or feeling overconfident? Could performance exceeding expectations be due to political decision makers compensating for the lack of preventative capacity through more stringent interventions, perhaps supported by geographical luck? All of this might have been a factor in the actual performance during the imminent crisis. We would, however, argue that it is wrong to solely put the blame or praise on the side of politics, when in all of these countries the decision-making was presented as evidence-based, and the GHSI purports to capture the whole range of what then seemed relevant, publicly available facts.

It is important to note that we have little evidence that the index actually formed a key part of policy-making in governments around the world, but that it is clear the index was constructed with this intention in mind. Given that the GHSI was obviously meticulously prepared, based on a wealth of data by a large group of international experts, we might even generalize the question to now ask whether the production of any such global composite indicator makes any sense at all as a strategic decision-making tool when faced with a global imminent crisis. In other words, is the intention of supplying a decision-support tool via a global index in time of an imminent health crisis realistic?

## Constructing a composite index

Initially, a composite index is simply a function of a series of underlying indicators. There are a number of steps and decisions involved in constructing good composite indicators. According to Mazziotta and Pareto [8] the following decision steps are crucial:

1. *Defining the problem!* For some issues there may exist a clear and agreed upon definition on how the problem is to be defined, and what its constitutive elements and basic categories are. The challenge here is to have a reliable system theoretic understanding which establishes nodes and contact points with other systems. With complex problems, such as the grand societal challenges, this step is often a topic of scientific dispute and even conflict. Regarding threats to health security in the face of pandemics, the choice of which factors and categories are crucial is an ongoing discussion.
2. *Selecting a group of individual indicators!* “Ideally, indicators should be selected according to their relevance, analytical soundness, timeliness, accessibility, among others. The selection step is the result of a trade-off between possible redundancies caused by overlapping information and the risk of losing information.” (ibid., p. 70). Again, a good insight into systemic interdependencies is necessary to make that choice. It involves judgement about the relative importance of individual indicators and avoiding those which are strongly correlated to others, since this increases redundancy. The indicators need to be measurable, i.e. they need concrete data to support them. Here, other issues arise; for example, when choosing the scale of measurement (e.g. national, regional, household etc.) or the numerical units of measurement (0 and 1, or a range of values between 0 and *n*?). If data are insufficient in one country, one may in certain cases apply Monte Carlo methods to impute missing data.
3. *Normalizing the selected indicators!* Here one has to make sure that comparability is achieved between different indicators among various categories, despite different measurement dimensions. Therefore, one transforms them to simple numbers, and those can be achieved by different means, for example, by ranking, distance to a reference point etc. This often has the effect of abstracting detail away, adding uncertainty to the accuracy of the number.
4. *Aggregating the normalized indicators!* This step combines the chosen indicators to just one or several aggregate indices. Since they are normalized, a simple method can be the mathematical addition of unit rankings. However, in complex situations one might face the need to weigh them differently, i.e. to assign weights to each indicator before they are aggregated into the final index to emphasize some indicators over others. Again, the assignment of weights is obviously a matter of subjective judgement, and we would surmise that this is often strongly biased by chosen value perspectives: “Different weighting techniques may be chosen, none of which is exempt by a discretionary choice” [9]. Furthermore, how are we to combine the different indicators? Most commonly this could be done by using weighted arithmetic mean values, but since this can imply that bad performance on one indicator can be countered by sufficiently good performance on others, it may not be adequate for what we want to measure in the combined index. One may then choose a weighted geometric mean for these values. There are formal methods to reduce the dependency on mere judgement, such as by assigning weight according to the variability of the indicator, since the greater the variability, we assume the greater its influence on what we try to measure with the index. However, even then our delineations will depend on an overall judgement.

In the scientific literature there are a number of contributions on how to improve the performance of composite indices, for instance in the area of food security, which admittedly is a very complex issue and approached from different angles (cf. [9–11]). There is significant progress in some of the technical issues involved in the construction of these indicators.

Yet, what one easily can infer from the above description is that building a composite index is not straightforward and involves a series of subjective judgments. It also involves dealing with system uncertainties and dealing with value presuppositions (cf. [12, 13]). Mazziotta and Pareto [8] express this the following way:

“No universal method exists for composite indices construction. In each case their construction is much determined by the particular application, including both formal and heurist elements, and incorporate some expert knowledge on the phenomenon.” ([8], p. 72).

One may ask then the following question: If the process to construct global composite indices is so complex and difficult, why do we have such a plethora of them around in the first place? The cynical answer would be because this is what scientists can do. The more neutral and probably more realistic answer would be because they communicate easily to decision makers. The typical policy question might be: ‘How are we doing in regard to X?’ where X is a rather complex societal problem, and then the follow up would be: ‘And how are we doing in regard to X compared to other countries?’ If we are able to provide a simple answer, preferably expressed as a number relation like a rank, then the decision maker regards this as highly useful, specifically when followed up by proposed measures on how to improve that number. The usual problem is that X as such is not dealt with in any singular scientific silo due to its complexity, but needs to be translated and broken down into subproblems which are conducive to the apparatus of the various scientific disciplines. This is a task involving trans-disciplinarity. Therefore, we concur that the intention of the construction of composite indices as planning tool is a valid one, but we also maintain that valuable information loss occurs on the way from the scientist to the decision maker. The information loss is often related to the implicit uncertainties or the implicit values because they become more invisible the closer and higher up one moves to the decision maker [14]. It is this information loss and simplification of outcome which severely restricts the use of a global index as a management tool in an imminent crisis situation.

We make the following observations in this regard:

1. Indices like the GHSI comprise several layers of specificity, with the aim to look for measurable (quantifiable) features that are considered essential for higher-level properties. By implication, it emerges that higher-level properties are not directly measurable, and that is why one seeks to circumvent the problem by using subordinated indicators which indirectly contribute to the higher-level property. Typically, these higher-level properties are not directly measurable due to their complexity, implying the possibility of diverse emergent and unpredictable phenomena. The issue is known in the literature as the subjectivity and contentious nature of identifying the very problem in the first place and then breaking down the problem in measurable subunits. Some global indices have successfully identified a simple list of parameters that people agree to be essential for the issue. A case in point is the Human Development Index [15], for which there has been wide agreement on the basic three individual indicators. In the case of the GHSI, biological preparedness was broken down into six categories with 34 indicators and 85 sub-indicators. However, while already the selection of the categories may be questioned, one may certainly also question the chosen indicators and sub-indicators. What comprises health security is debatable and a matter of subjective judgement. Implicit in the choice are problematic questions like whether or not some indicators are substitutable or non-substitutable. Can, for instance, wide social compliance with protective measures and good risk communication reduce the number of necessary intensive care beds?
2. The upward process from the concrete to the more abstract implies building uncertainties upon uncertainties, without the means to precisely quantify these uncertainties. In global studies, large uncertainties are typically already present through differences in how base local data are registered and counted. Communicating a single index score or rank for each country masks the inherent uncertainty and volatility in the measurements. While statistical methods exist to, for example, combine uncertainty with sensitivity analysis [10], they do not eliminate deep uncertainty but simply transfer the uncertainties into quantifiable units [11].
3. Groups of properties that fall under a common concept are presumed to be uniformly linear and additive when contributing towards a common concept. This excludes local variation in response to the threat/property indexed in the study. One such variation might be cultural differences in risk communication. This approach also ignores interdependencies and mutual strengthening or weakening of constitutive properties. Typically, one tries to circumvent this by normalization of the various indicators to make them comparable to each other. While the process of normalization can be done in various ways, it will by necessity involve some subjective judgement on systemic dependencies.
4. Constructing a global composite index as a strategic tool in decision-making presupposes the existence of a normative benchmark for ideal states. Any such benchmark will indirectly introduce a socio-political and cultural bias that does not do justice to the diversity of viewpoints among both experts and non-experts. Countries with high compliance to imposed rules and regulations may have other needs in terms of preparedness than countries with low compliance. This point could be less important if the users have the time to critically assess the method the index is built on, in particular the match of the underlying problem (in our case, the preparedness to health threats) and the chosen indicators with their local conditions. However, in times of an imminent crisis one must assume that each crisis provides its own complex challenges, and the initial efforts are focussed on understanding the particular nature of the threat. For instance, the kind and speed of transmission, the need for protective gear (PPE), contact tracing methods, availability of intensive care units or the possibility and accessibility of vaccination emerged gradually as assessment tasks while the current crisis of Covid-19 already was a fact.
5. While solid and comprehensive reporting of the way a global composite indicator was constructed may shield one from some academic criticism, the fact remains that users of the index, the policy makers, will almost certainly focus on the overall performance figures as reflected in the indicator. In the current example, the authors of the GHSI stated that “national health security is fundamentally weak around the world” and that “no country is fully prepared for epidemics or pandemics, and every country has important gaps to address” [3]. But that did not stop a Member of Parliament in the South African National Assembly from claiming “the Global Health Security Index for 2019 Report revealed that South Africa is ranked 34 out of 195 countries… this gives confidence that the South African government through the national Department of Health is doing all within its power to strengthen its health systems to safeguard the public from the outbreak of any other form of infections” [16]. The key utility of a global index is ultimately being able to rely on the overall performance ranking.
6. Breaking down a politically important property into its sub-indicators and other elements always runs the risk of leaving out system interactions and interdependencies which are not routinely assessed in specific academic disciplines. For instance, while the coronavirus pandemic in most countries activated epidemiologists and virologists, in some countries it also activated psychologists, economists, social scientists and philosophers. Thus, there is bias already in the framing of the issue, and alternative framings that would emerge in a transdisciplinary approach are seldom considered.

It is by no means surprising that scientific systematization will always include uncertainties and will never be completely objective. Facts and values are intertwined in science for policy. The framework of post-normal science [12, 13] has stressed this for a long time. It has also been pointed out in areas outside of health that composite indices may be misleading and may hide important information in some of its elements. Giampietro and Saltelli [17] have raised this issue in regard the global ecological footprint, and this has spurred a number of reactions [18–20]. We also note other composite indices that rely on poor proxies [8, 11], such as the Organisation for Economic Co-operation and Development (OECD) Better Life Index [21] or university rankings [22]. The danger is that policy decisions based on flawed indices and rankings are likely to be equally flawed.

## What might be better?

Let us put the question the other way round: could one make sensible and scientifically informed policies without these global indicators or index? With the experience of Covid-19 fresh in our minds, we would venture that good pandemic policies (leaving out the other issues for the time being) could be based on and started with sensible data presentation and some simple heuristics rather than over-stated modelling with its inherent limitations. One key to effective control of the pandemic was acting preventatively at an early stage, and implementing counter-measures such as widespread testing, lockdown and closing of the borders [23]. Taiwan is one of the best examples in this respect: noting the rapid rise of infections in neighboring China in late December 2019, it implemented wide-spread testing among incoming people, and set in motion a National Health Command Center. It soon closed its borders, quarantined all cases and rapidly propagated the use of face masks. Taiwan certainly did not find any advice in the GHSI since it was not included in the first place. Early detection and reaction were the key to controlling the pandemic in many countries, and they showed success. Laissez-faire attitudes like in the United Kingdom, Sweden or the United States of America proved fatal. A United Nations report has this key message: “Act decisively and early to prevent the further spread or quickly suppress the transmission of Covid-19 and save lives” [24]. Other writers have already noted that simplicity may be a better guide than getting lost in the complexities: “An imperative to prioritize simplicity over complexity is at the core of social health” [25].

Furthermore, in all modelling it is widely recognized that there is trade-off between precision and complexity. Complex models are seen as more accurate, while simple ones are seen as more general with a lack of detail that causes systematic bias in predictions—but adding detail to a model does not guarantee an increase in reliability unless the added processes are essential, well understood and reliably estimated [26]. O’Neill’s conjecture was that there may be an optimal balance between model complexity and model error ([26], p. 70). In our case, this implies that adding to the complexity of the basic categories in the GHSI may actually increase model error rather than decrease it. This is also the background for the recommendations in [27]. To quote this article:

“Complexity can be the enemy of relevance. Most modelers are aware that there is a tradeoff between the usefulness of a model and the breadth it tries to capture. But many are seduced by the idea of adding complexity in an attempt to capture reality more accurately. As modelers incorporate more phenomena, a model might fit better to the training data, but at a cost. Its predictions typically become less accurate. As more parameters are added, the uncertainty builds up (the uncertainty cascade effect), and the error could increase to the point at which predictions become useless.” ([27], p. 483).

Here we want to stress that we are concerned with meeting an immediate health crisis and we ask whether or not an index like the GHSI can be regarded as a useful addition in our toolbox to manage that crisis. In the preceding sections, we have already claimed that as a matter of fact it was not useful, and certainly was not a precise predictor. But the real underlying question we want to ask is if we are looking in the wrong toolbox altogether. As with all tools, the utility of the tool depends on its intended use. Therefore, we do not argue that there is no use for a composite index like the GHSI, since we might assume that it could have good uses as a tool in the design of long-term strategies in our health policies. What we, however, claim, is that policy in an imminent crisis like a pandemic is ill advised if it looks at the composite index as a guide to crisis management. This does not imply that science cannot contribute to crisis management, rather the opposite: science is highly useful if the right information comes in the right format and the right doses at the right times. It only implies that scientific advice may take other roads to policy than a global composite index. One issue might be to resist the temptation to provide numbers, i.e. quantification, when the problem is still poorly understood.

“Quantification can backfire. Excessive regard for producing numbers can push a discipline away from being roughly right towards being precisely wrong.” ([27], p. 484).

One needs to recognize the immediate needs of decision makers facing an immediate crisis. Obviously, a decision maker tries to come up with robust decisions, and robust decisions are typically about a set of different future scenarios under deep uncertainty and guided by varying criteria for robustness; for example, a decision maker may change from an optimist to a pessimist strategy or the other way round [28, 29]. Decision makers need to engage in a learning process as the crisis unfolds, and thus apply an adaptive management approach [30] through the different phases of the crisis. The availability of a risk register may be a decision-support tool during this process. As the science–policy interface is known to be full of pitfalls, institutionalized brokerage may be an important support [31–34], aiming at synthesis when information is sparce and beset with deep uncertainty. Heuristics may be more important than formal tools, aiming at characterizing the whole complexity of the issue at hand. As Todd and Gigerenzer [35] observe, simple heuristics often perform comparably or better than more advanced algorithms, and they add a much-desired simplicity which leads to more robust decisions. This point does not invalidate some other uses of quantified formal indices or models, but it stresses that the scientific input needs to meet the constraints and context of the decision situation in a crisis.

In such a setting, the availability of reliable data on the emergence of the risk is typically providing a good input for decision heuristics. We illustrate this by reference to our INGSA Evidence-to-Policy Tracker.

## Conclusions

Based on our INGSA Evidence-to-Policy Tracker project [https://www.ingsa.org/covid/policymaking-tracker-landing/], we have been able to analyse the interventions taken by over 120 countries and when they took place. From these data, we have seen two particular patterns so far. In East Asian countries, such as Japan and South Korea, governments took swift action to increase the supply of PPE and face masks, and began public education campaigns at a very early stage—at least 14 days before the third death, thereby avoiding the need for harsh restrictions. In some other developed countries, lockdown measures, such as limiting gathering sizes, closing schools, limiting non-essential movement and closing borders, were implemented well before the threat of Covid-19 spiraled out of control. The countries that have fared the worst in terms of deaths per million people waited longer before implementing similar policies, as shown in Table 2. A similar analytical approach was taken by journalists at POLITICO when comparing lockdown measures across Europe [36]. It should be noted that a number of countries with fragmented state-level responses were not included in Table 2. A pattern like this obviously does not capture the complexity of the situation; for example, it does not incorporate the implementation or enforcement of these policies, nor does it distinguish between policies at different scales. And there are always exceptions, but the simple heuristic of acting preventatively and quickly is visible in the data.

**Table 2:**
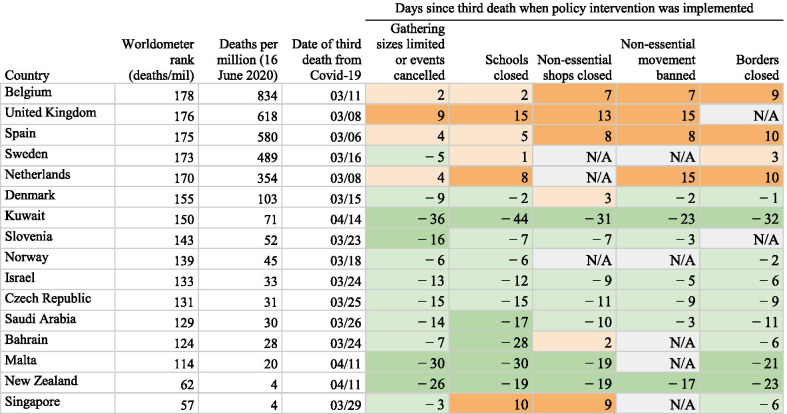
A selection of countries and policies, highlighting the number of days each policy was implemented since the third death from Covid-19.

The table is sorted by the Worldometer Rank based on the number of deaths per million people. N/A indicates that this policy had not yet been implemented in that country as of 16 June 2020

Based on the analysis presented in the preceding sections, we hypothesize that there are some decision steps that may serve as useful and simple heuristics for performing well in a pandemic:

1. Recognize the threat to your country and the need for a response early
2. Agree on a broad and transparent societal basis what response strategy is most acceptable for your country, such as elimination of the virus within your borders, “flattening the curve”, or keeping the occurrence of infections below a predefined damage threshold
3. Fill the chosen response strategy with a combination of practical measures appropriate to the epidemiological, economical, and socio-cultural circumstances, and monitor their effectiveness as the crisis unfolds
4. Adjust, or change your chosen strategy according to the predefined goals and current data, preferably by inclusion of various sources of expertise and good communication with society.

Through further analysis of the INGSA Evidence-to-Policy Tracker data, we aim to understand the role of timing of interventions, the levels of responses and the sources of evidence and justification behind these approaches. This will allow us to categorize the different types of strategies taken by governments around the world and identify different styles of leadership and ideological underpinnings. It is noteworthy that the most obvious control mechanism successfully used, border closure, was not seen by the WHO as a key response, yet border closure was key to the elimination of COVID-19 in island states where early closure was perhaps easier. However, there is likely no global silver bullet to avoid or contain an emerging pandemic. Realizing the diversity of values along with epidemiological, economic and cultural considerations into a robust strategy is also a bridge to societal compliance.

## Keep it simple

We conclude with the hypothesis that in order to be prepared for pandemics or manage other imminent crises, we may not need more sophistication in the construction of global composite indicators. In these situations, these indices may not be as useful as they purport to be. We may actually do without them to a large extent and could learn from the past instead, recognizing the power of simple heuristics that make sense for the context and point us in the right direction. We may still consult global indices with a critical spirit and deep understanding of their inherent assumptions when working out long-term strategies for improvements in various sectors of policy. Yet, in a pandemic like the current one we see simple heuristics and adaptive management as key to robust policies.

## Data Availability

The data sets generated during and/or analysed during the current study are available in the INGSA COVID-19 Evidence-to-Policy Tracker repository, https://www.ingsa.org/covid/policymaking-tracker-landing/; the Worldometer COVID-19 Coronavirus Pandemic repository, https://www.worldometers.info/coronavirus/?; and the 2019 Global Health Security Index, https://www.ghsindex.org/#l-section--map.

## Abbreviations

GHSI: Global Health Security Index
INGSA: International Network for Government Science Advice
WHO: World Health Organization

## Footnotes

1 However, a quick glance at the rankings of the six categories can already convey that no radical changes can be expected that would bring the combined ranking closer to the Worldometer data.

## References

[1] Cameron EE, Nuzzo JB, Bell JA, et al. Global Health Security Index. Building collective action and accountability. Nuclear Threat Initiative & Johns Hopkins Center for Health Security. October 2019. https://www.ghsindex.org/#l-section--map. Accessed 19 June 2020.

[2] Phelps G, Crabtree S. Worldwide, Median Household Income about $10,000. Gallup. 2013. https://news.gallup.com/poll/166211/worldwide-median-household-income-000.aspx. Accessed 19 June 2020.

[3] Johns Hopkins University (Hub). Here's the Johns Hopkins study President Trump referenced in his coronavirus news conference. 28 February 2020. https://hub.jhu.edu/2020/02/27/trump-johns-hopkins-study-pandemic-coronaviruscovid-19-649-em0-art1-dtd-health/. Accessed 19 June 2020.

[4] Aitken T, Chin KL, Liew D, Ofori-Asenso R. Rethinking pandemic preparation: Global Health Security Index (GHSI) is predictive of Covid-19 burden, but in the opposite direction. J Infect. 2020;81(2):318–56 10.1016/j.jinf.2020.05.001. 10.1016/j.jinf.2020.05.001 PMC7207133 32437727

[5] Abbey EJ, Khalifa BAA, Oduwole MO, Ayeh SK, Nudotor RD, Salia EL (2020). The Global Health Security Index is not predictive of coronavirus pandemic responses among Organization for Economic Cooperation and Development countries. PLoS ONE.

[6] Razavi A, Erondu NA, Okereke E (2020). The Global Health Security Index: what value does it add?. BMJ Glob Health.

[7] Chang CL, McAleer M (2020). Alternative global health security indexes for risk analysis of Covid-19. Int J Environ Res Public Health.

[8] Mazziotta M, Pareto A (2013). Methods for constructing composite indices: one for all and all for one. Riv Ital Econ Demogr Stat.

[9] Santeramo FG (2015). On the composite indicators for food security: Decisions matter!. Food Rev Int.

[10] Caccavale OM, Giuffrida V (2020). The Proteus composite index: towards a better metric for global food security. World Dev.

[11] Saltelli A, Ratto M, Andres T, Campolongo F, Cariboni J, Gatelli D, et al. Global sensitivity analysis. The primer. Chichester: Wiley; 2008. https://onlinelibrary.wiley.com/doi/book/10.1002/9780470725184.

[12] Funtowicz SO, Ravetz JR (1990). Uncertainty and quality in science for policy.

[13] Funtowicz SO, Ravetz JR (1993). Science for the Post-Normal Age. Futures.

[14] Kaiser M. “On Scientific Uncertainty and the Precautionary Principle”. In: Gethmann CF, Carrier M, Hanekamp G, Kaiser M, Kamp G, Lingner S et al., editors. Interdisciplinary research and transdisciplinary validity claims. Berlin Heidelberg: Springer; 2015. p. 138–58. https://www.researchgate.net/profile/Stephan_Lingner/publication/268149000_Interdisciplinary_Research_and_Trans_disciplinary_Validity_Claims/links/54623db80cf2c0c6aec1ac99.pdf. Accessed 19 June 2020.

[15] United Nations Development Programme (UNDP) (2010). Human development report 2010.

[16] Parliamentary Monitoring Group. Unrevised Hansard for Proceedings of the National Assembly. 5 March 2020. https://pmg.org.za/hansard/29979/. Accessed 19 June 2020.

[17] Giampietro M, Saltelli A (2014). Footprints to nowhere. Ecol Ind.

[18] Goldfinger S, Wackernagel M, Galli A, Lazarus E, Lin D (2014). Footprint facts and fallacies: a response to Giampietro and Saltelli (2014) “Footprints to Nowhere”. Ecol Ind.

[19] Galli A, Giampietro M, Goldfinger S, Lazarus E, Lin D, Saltelli A (2016). Questioning the ecological footprint. Ecol Ind.

[20] Van Den Bergh J, Grazi F (2010). On the policy relevance of ecological footprints. Environ Sci Technol.

[21] Organisation for Economic Co-operation and Development (OECD). OECD Better Life Index. 2020. http://www.oecdbetterlifeindex.org/. Accessed 19 June 2020.

[22] Shin JC, Toutkoushian RK, Teichler U (2011). University rankings: theoretical basis, methodology and impacts on global higher education.

[23] Lu N, Cheng K-W, Qamar N, Huang K-C, Johnson JA (2020). Weathering Covid-19 storm: successful control measures of five Asian countries. Am J Infect Control.

[24] United Nations. Shared responsibility, global solidarity: responding to the socio-economic Impacts of Covid-19. March 2020. https://unsdg.un.org/sites/default/files/2020-03/SG-Report-Socio-Economic-Impact-of-Covid19.pdf. Accessed 19 June 2020.

[25] Hobsbawn J. Simplicity, clarity and minimalism: social health during Covid-19. OECD Forum. 2020. https://www.oecd-forum.org/users/389156-julia-hobsbawm/posts/66165-simplicity-clarity-and-minimalism-social-health-during-covid-19. Accessed 19 June 2020.

[26] Turner MG, Gardner RH. Introduction to models. In: Turner MG, Gardner RH, editors. Landscape ecology in theory and practice. New York: Springer; 2015. p. 63–95.

[27] Saltelli A, Bammer G, Bruno I, Charters E, Di Fiore M, Didier E (2020). Five ways to ensure that models serve society: a manifesto. Nature.

[28] Popper SW (2019). Robust decision making and scenario discovery in the absence of formal models. Futures Foresight Sci.

[29] Giuliani M, Castelletti A (2016). Is robustness really robust? How different definitions of robustness impact decision-making under climate change. Clim Change.

[30] McLain RJ, Lee RG (1996). Adaptive management: promises and pitfalls. Environ Manag.

[31] Todd PM, Gigerenzer G (2000). Précis of “Simple heuristics that make us smart". Behav Brain Sci.

[32] Organisation for Economic Co-operation and Development (OECD). “Scientific advice for policy making: the role and responsibility of expert bodies and individual scientists”. OECD Science, Technology and Industry Policy Papers, No. 21. Paris: OECD Publishing. 10.1787/5js33l1jcpwb-en. Accessed 19 June 2020.

[33] Gluckman P (2014). Policy: the art of science advice to government. Nature.

[34] Gluckman P (2017). Enhancing evidence-informed policy making.

[35] Gluckman P (2018). The role of evidence and expertise in policy-making: the politics and practice of science advice. J Proc R Soc NSW.

[36] Hirsch C. Europe’s coronavirus lockdown measures compared. POLITICO. 2020. https://www.politico.eu/article/europes-coronavirus-lockdown-measures-compared/. Accessed 19 June 2020.

